# The measurement, evolution, and neural representation of action grammars of human behavior

**DOI:** 10.1038/s41598-021-92992-5

**Published:** 2021-07-02

**Authors:** Dietrich Stout, Thierry Chaminade, Jan Apel, Ali Shafti, A. Aldo Faisal

**Affiliations:** 1 Department of Anthropology, Emory University, Atlanta, GA USA; 2 Institut de Neurosciences de La Timone, Aix Marseille Université, Marseille, France; 3 Department of Archaeology, Stockholm University, Stockholm, Sweden; 4 Department of Bioengineering, Imperial College London, London, UK; 5 Department of Computing, Imperial College London, London, UK; 6 Integrative Biology, MRC London Institute of Medical Sciences, London, UK; 7 Behaviour Analytics Lab, Data Science Institute, London, UK

**Keywords:** Archaeology, Cognitive neuroscience, Human behaviour

## Abstract

Human behaviors from toolmaking to language are thought to rely on a uniquely evolved capacity for hierarchical action sequencing. Testing this idea will require objective, generalizable methods for measuring the structural complexity of real-world behavior. Here we present a data-driven approach for extracting action grammars from basic ethograms, exemplified with respect to the evolutionarily relevant behavior of stone toolmaking. We analyzed sequences from the experimental replication of ~ 2.5 Mya Oldowan vs. ~ 0.5 Mya Acheulean tools, finding that, while using the same “alphabet” of elementary actions, Acheulean sequences are quantifiably more complex and Oldowan grammars are a subset of Acheulean grammars. We illustrate the utility of our complexity measures by re-analyzing data from an fMRI study of stone toolmaking to identify brain responses to structural complexity. Beyond specific implications regarding the co-evolution of language and technology, this exercise illustrates the general applicability of our method to investigate naturalistic human behavior and cognition.

## Introduction

For more than 60 years, the serial ordering of behaviour has been a core topic for the cognitive and behavioral sciences^1,2^. Enhanced capacities for complex action sequencing support distinctive human behaviors such as language^3^, imitation^4^, and tool use^5,6^, and are fundamental to the flexibility that is a hallmark of human intelligence^7,8^. It has been suggested that this implies a unitary neurocognitive foundation for human behavioral uniqueness across domains^1,5,6^, but this remains controversial^9^. Although theory^1^ and modelling^10^ suggest computational similarities across behaviours ranging from tool-use and foraging to language learning, empirical investigation outside domains with established notational systems (language, music, mathematics) has been limited by a lack of objective, generalizable methods for describing, quantifying, and comparing the sequential structure of diverse, real-world behaviours.

Research on motor sequence^11–13^, implicit^14,15^, and statistical learning^16^ has provided evidence of underlying neural and cognitive mechanisms, but has generally been limited to highly artificial tasks such as executing invariant key-press sequences or recognizing simple artificial grammars of known structure. This limits our ability to generalize findings to understand the learning and execution of the real-world skills^17,18^ of interest to fields ranging from sport science^19^ and surgery^20^, to human behavioral ecology^21^, and comparative psychology^22^.

In human origins research specifically, investigation of long-standing hypotheses about the evolutionary relationships between tool making, language, and cognition have been hampered by the lack of an objective metric for the behavioural complexity of different ancient human technologies^23–26^. Here we adopt a data-driven computational approach to this challenge by using grammatical pattern recognition algorithms to measure the structural complexity of behavioral sequences from modern tool-making replication experiments—effectively extracting action grammars for critical survival skills from the human evolutionary past. This allows us to isolate and compare the structural complexity of “noisy” natural behaviors that simultaneously vary across a wide range of other perceptual, motor, and kinematic dimensions, including identification of specific brain responses to this complexity.

**Figure 1:**
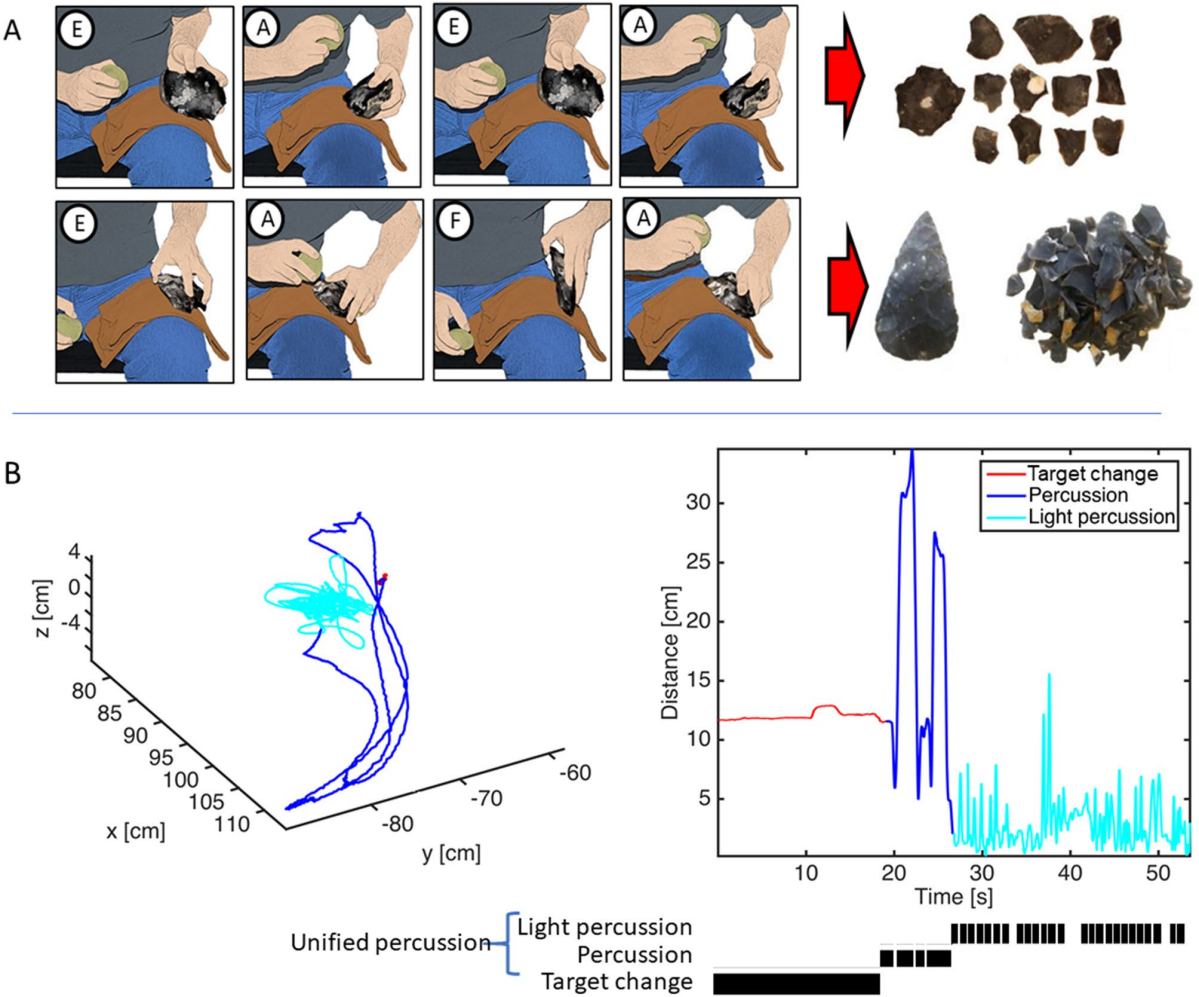
Symbolic Modeling of tool-making action sequences. (**A**) Oldowan (top) and Acheulean (bottom) action sequences were coded using 7 event codes (circled letters, see Materials & Methods). Products illustrated to the right, individual depicted is the first author. (**B**, Left) Spatial trajectory of the hammer stone during toolmaking. The X–Y plane is aligned with the plane of the percussion strike hand-arm movements. The core is centred at approximately 80 cm, − 70 cm, 0 cm. (**B**, Right) Distance of the hammerstone from the centre of the core. Target change movements are coded in red. Percussion strike trajectories are in dark blue, light percussion movements (here for platform preparation) are in light blue. Below, the black bars indicate the corresponding timing and duration of the ethogram.

**Figure 2:**
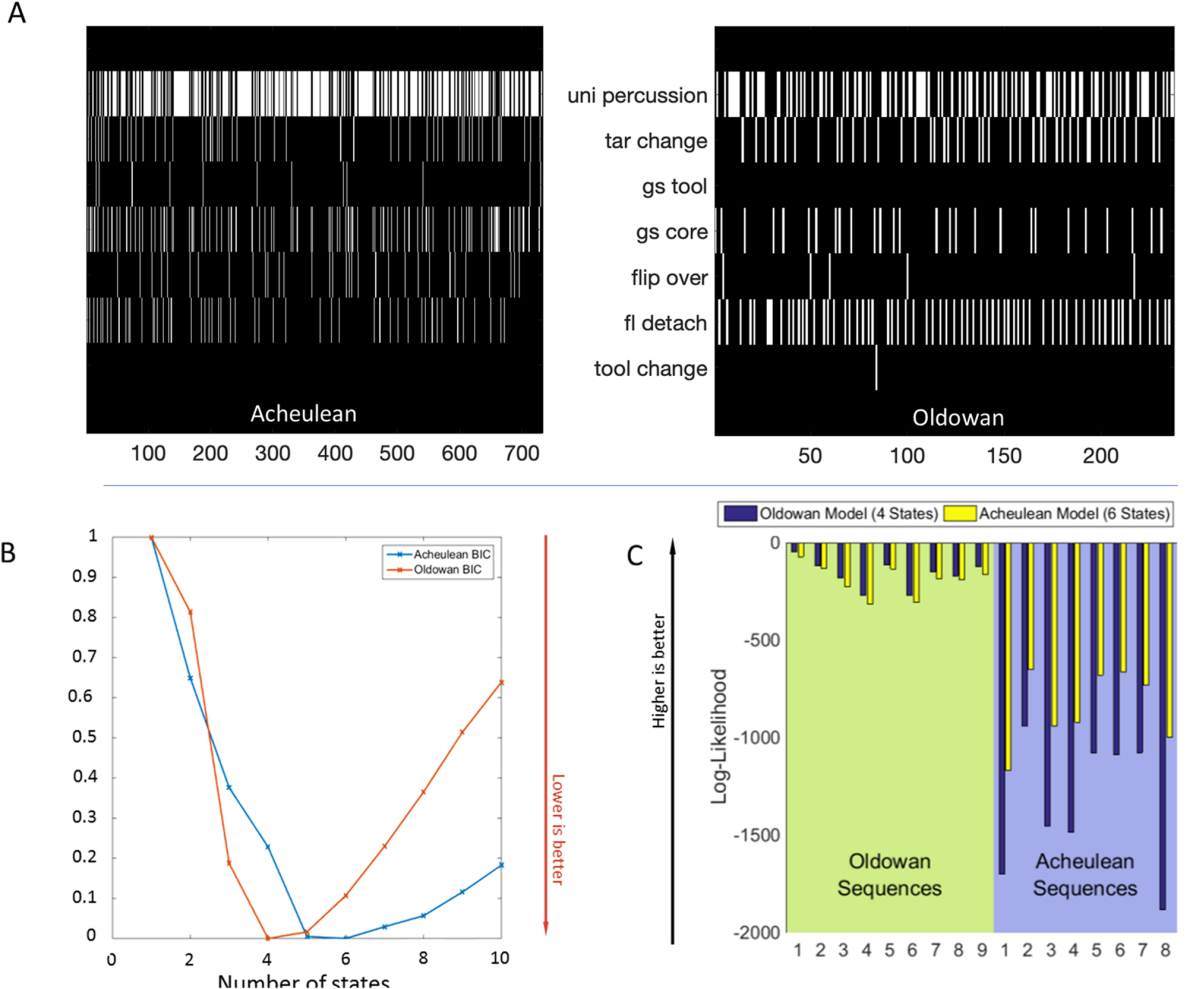
(**A**) Raster plot of two typical Acheulean (left) and Oldowan (right) tool making sequences. Each row in the raster plot represents the observation of one of the 7 actions. The symbolic representation has been abstracted from explicit timing information (cf. Fig. 1.B) to a time-scale invariant sequence representation that counts sequence position. (**B**) Bayesian Information Criterion values (less is better) across models with increasing numbers of hidden states. Red, Oldowan, Minimum = 4; Blue, Acheulean, Minimum = 6. (**C**) Log-Likelihood values indicating model fit (higher is better) across sequences. Fit for Oldowan sequences is better overall; Acheulean model fit to Oldowan data is better than Oldowan model fit to Acheulean data.

We conducted 17 tool-making replication experiments and coded the behavior sequences that were generated (Fig. 1A,B). This sample includes 5 sequences for which upper limb movements and manual joint angles were recorded as part of a previous study^27^, and 6 for which the tools and waste produced were analyzed and compared with actual Paleolithic artifacts from the Middle Pleistocene site of Boxgrove, UK^28^. Building on this and other previous research^29–32^, we focused our current study on archaeologically documented tool-making methods from the early and late Lower Paleolithic, a period that witnessed a nearly threefold increase in hominin brain size. This allows us to empirically address the over 100 years of theorizing linking increasingly complex tool-making with brain evolution and language origins^6,33–35^. The early (Oldowan, ca. 2.5 Mya) technology modeled here comprised the production of simple, sharp-edged stone flakes by striking one stone with another. The late (Late Acheulean, ca. 0.5 Mya) technology comprised the production of refined, teardrop-shaped “handaxes” through intentional shaping. We defined a shared action alphabet, consisting of 7 event types encompassing the elementary body movements and object transformations present in every sequence of both technologies (Fig. 2A), and applied two established sequence learning algorithms to the coded event sequences: Hidden Markov Models (HMM) and k-Sequitur.

## Results

### Hidden Markov modeling

HMM detects probabilistic regularities (hidden states) across sequences and can capture the structure of arbitrarily complex sequences given sufficient numbers of hidden states. The optimal number of hidden states provides a measure of structural complexity. We fitted HMMs to coded event sequences and computed the Bayesian Information Criterion (BIC) across different numbers of hidden states as a measure of model fit. BIC reached its minimum (less is better) at 4 hidden states for Oldowan and 6 for Acheulean data (Fig. 2B), indicating a 50% increase in complexity. These two models perfectly categorized the sequences (likelihood greater for correct model, Fig. 2C). The fit was better for both models on the simpler Oldowan sequences. The close fit of the Acheulean model to Oldowan data (but not vice versa) indicates that the former captures most of the structure of the latter, and that Oldowan sequences may be considered a subset of Acheulean sequences.

**Figure 3:**
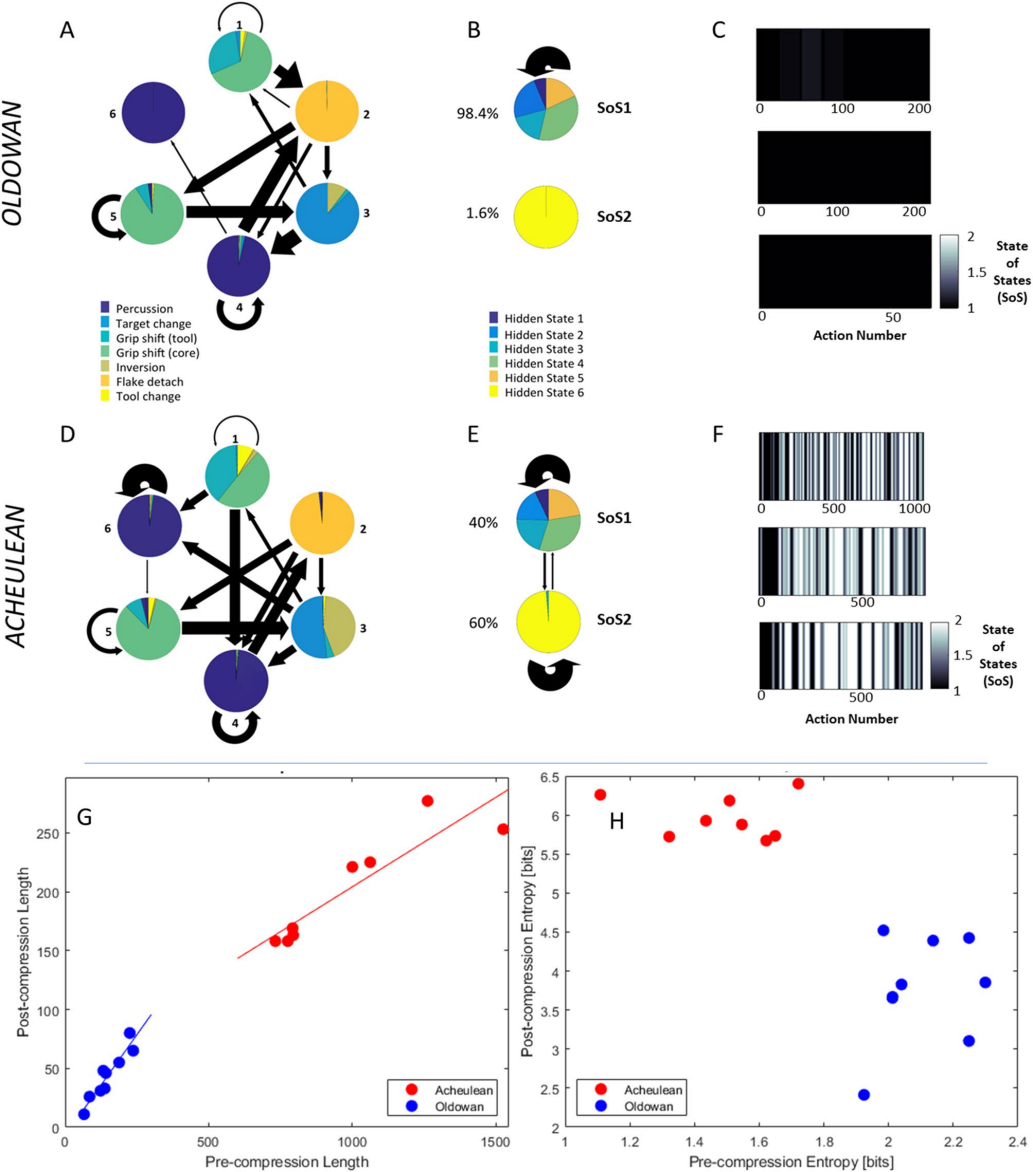
Results of grammatical analysis: HMM analysis (**A**–**F**) Empirical transition (arrows) and emission (pie charts) matrices of 6-state HMM fitted to all Oldowan (A) and Acheulean (D) sequences. Arrow thickness indicates transition probabilities between states (values < 5% not displayed). Pie chart area indicates probability of an action being performed in that state. Oldowan Hidden State 6 accounts for less than 2% of all data points. In the middle are similar illustrations of the superordinate "States-of-States” for Oldowan (**B**) and Acheulean (**E**) data. At right are examples of the running average State of States for Oldowan (**C**) and Acheulean (**D**) time-series. Black: everything in SoS 1; white: everything in SoS 2. Sequitur analysis (**G**,**H**): Effect of Sequitur compression on Acheulean (red) and Oldowan (blue) sequences, show clear differences between Oldowan and Acheulean sequences as measured by pre- and post-compression length (**G**). Note, how the slope of pre/post compression straight line fits is clearly different between Oldowan and Acheulean. Similarly, the information content within the uncompressed and compressed sequences as measured is clearly different (**H**).

We therefore used the Acheulean HMM to test for further structure. We obtained the most likely hidden state sequences for the Oldowan and Acheulean data and then fitted a second, 2-state HMM onto these higher-order sequences (Fig. 3). We found that Acheulean sequences oscillate between two superordinate states-of-states (SoS) whereas Oldowan sequences remain in one). Thus, Acheulean sequences display an additional level of structure not expressed by Oldowan sequences.

Next, we fit the 6-state Acheulean HMM to Oldowan and Acheulean data and observed the probability of actions per hidden state as well as transitions between hidden states. Our Oldowan data are characterized by the repetition of one simple action “chunk” consisting of a relatively invariant sequence of states (3- > 4- > 2: Fig. 3A) that essentially corresponds to the removal of an individual flake and is entirely captured by SoS1 (Fig. 3B,C). Acheulean sequences are more variable (Fig. 3D), reflecting the addition within some flake removal chunks of a sub-operation archaeologists refer to as striking platform preparation. This involves repeated low-amplitude (see “Methods” section and Fig. 1B) chipping of striking surfaces to alter their sharpness, bevel, and placement relative to the midline. This operation is captured at the next level by SoS2 (Fig. 3E). SoS2 is less frequent in the early stages of our sequences (Fig. 3F) which is consistent with the presence of an initial “roughing out” stage in handaxe manufacture prior to more refined shaping^23^. Introspection by experienced tool-makers^32,36^ has previously suggested that platform preparation increases the complexity to Paleolithic action organization, but it has not previously been possible to test this intuition objectively or to quantify the magnitude of increase in a generalizable way. Our HMM method thus captures meaningful (i.e. goal directed) regularities in stone tool-making in a data-driven way that: 1) derives structure rather than imposing it, 2) respects the real variability underlying ideal characterizations, 3) enables objective quantification of grammatical complexity, and 4) is readily adaptable to the study of other sequential behaviors.

The Chomsky hierarchy in Formal Language Theory describes a series of increasingly powerful and inclusive computational systems, or grammars, differentiated by their memory resources^1,37^. A simple Markov chain is a memoryless probabilistic system equating to a regular (finite-state) grammar that does not permit long distance dependencies. HMMs are dynamic Bayesian networks that asymptotically approximate supra-regular context-free grammars (with unbounded memory) through the progressive addition of hidden states. The increase we observed in the optimal number of hidden states from Oldowan to Acheulean thus provides a measure of increased grammatical complexity and memory requirements without positing infinite capacity. This modeling approach is consonant with the view that finite-state, probabilistic, and parallel computational models are cognitively and neurobiologically realistic^7,37–39^. Others, however, contend that human cognition is in fact characterized by constitutively hierarchal processing using supra-regular resources, that humans have a tendency to employ such context-free solutions even when they are not actually necessary^1^, and that such tree-based algorithms are more efficient for learning^40^.

### Context-free grammar fitting

We therefore pursued a second approach by fitting context-free grammars (CFGs) to the tool-making sequences. Formal Language Theory employs terminal symbols (in our case 7 event types) and non-terminal symbols (re-write rules expandable to terminal and/or non-terminal symbols) to generate strings. Ultimately, we have to solve the minimal grammar problem, i.e., finding the unknown grammatical rulebook that provides the simplest explanation given the observed sequence data. Whereas regular grammars and HMMs are driven by local relationships between symbols, CFGs capture nested dependencies of theoretically infinite length and depth. The standard algorithm to extract deterministic CFGs, Sequitur^41^, creates a new rule as soon as a terminal symbol pair is observed twice in a sequence and repeats this pair-wise aggregation, adding new levels of superordinate rules until the complete sequence is described. This makes Sequitur powerful but liable to detect a high number of spurious (occurring < 3 times) rules in the variable sequences generated by real human behavior. We therefore advance a simple variant of Sequitur, k-Sequitur, which requires a pair to occur k-times before generating a rule. Increasing k makes the grammar discovery process less sensitive to infrequent pairs and less prone to creating rules from noise. Nevertheless, both sequitur (2-sequitur) and higher-k versions produce the same qualitative results (see Supplementary Fig. S1):

In agreement with our HMM results, deterministic CFG extraction found Oldowan grammars to be a less complex sub-set of Acheulean grammars. Rule inference from combined Oldowan and Acheulean samples identified multiple rules that occur only in Acheulean sequences (Supplementary Fig. S2) and showed that the frequency of Acheulean-only rules increases at higher levels (0 at level 2, 1 at Level 3, 2 at Level 4, 5 at Level 5). No Oldowan-only rules were identified, even when rule inference was restricted to the Oldowan data set. CFG extraction achieved substantial compression of both Oldowan and Acheulean sequences (Fig. 3G), however the rate (inverse slope) of Acheulean compression was more than twice as great (7.69 vs. 2.94). This indicates that Acheulean sequences have more structure for rule-based compression, in an approximate 2:1 ratio paralleling our HMM finding of two Acheulean SoS vs. one Oldowan. Each post-compression Acheulean element (rule or terminal symbol) contains more information (measured as Shannon entropy: Fig. 3H), yet Acheulean grammars still require more non-terminal symbols (rules) to achieve a comparable fit to the data. These compression results are robust over increasing k values (k = 4, k = 8; see Supplementary Fig. S1). In addition to our bit-based representation of absolute entropy change as a result of compression, we also considered normalized entropy to ensure comparability. For this, each entropy value is divided by the theoretical maximum entropy, relating to a case of equiprobable states (see Supplementary Fig. S3). CFGs can parse regular strings, so fitting CFGs to our sequences in this way does not imply that supra-regular resources are required. It does show that the greater complexity and depth of Acheulean sequences is robust even assuming such resources.

Our CFG results reveals that the greater complexity of Acheulean sequences is due to long strings of repeated percussions, the removal of which eliminates Oldowan/Acheulean differences in compression rate (Supplementary Fig. S4). These strings comprise the same repeated, low-amplitude chipping of striking platforms (Fig. 1B) extracted as SoS2 in our HMM analysis and corresponding to the tool-making operation known as platform preparation^28^. HMM and CFG methods thus converge, not only to quantify the greater complexity of Acheulean sequences, but also to extract a key technological element of the instrumental structure of Acheulean tool making that largely accounts for this difference.

## Discussion

The results show that our grammar extraction methods are able to discover the instrumental structure of behavior directly from the structure of action sequences coded using a minimalistic and objective ethogram, without requiring subjective functional or intentional interpretations by the observer. These methods are easily generalizable to other behaviors and, in the specific case of Paleolithic tool making, provide new means to investigate the archaeological record of technological change. By using a single elementary action alphabet, we can consider variation within as well as between putative behavior categories in strictly equivalent terms, treating variation as a source of information rather than noise^42^. This provides a method for studying the structure and neurocognitive foundations of complex and variable real-world behaviors in a way that complements ongoing research into structured sequence learning that uses artificial experimental tasks.

To date, motor sequence learning research has generally employed simple sequences of basic motor actions (e.g. finger movements, key-presses) to address questions about the time-course and neural mechanisms of learning (e.g.^12,13,43^). Serial Reaction Time Tasks^15^ add a sensory element by making responses contingent on a (usually visuospatial) cue. This enables study of more complex sequences, such as "high-order" (n-gram, n > 1) Markov chains, to address additional questions including the contributions and neural foundations of explicit vs. implicit learning^44^. This has also been a focus in the statistical learning literature, which has classically employed artificial grammar learning^14,16^ paradigms to investigate discrimination (indicated by button press or looking time) of valid vs. violation sequences. Among other things, this work has demonstrated the relevance of statistical learning to human language comprehension^16,45,46^ and explored the language-relevant capacities of nonhumans^40,47,48^.

Despite the quality and quantity of research on motor and statistical learning, however, important questions remain to be addressed. For example, it is controversial whether structured sequencing is supported by a single, domain-general mechanism^49–51^ or by parallel computations in multiple, modality- or task-specific systems^9,52^. Similarly, it remains unclear to what extent mechanisms for sequence perception overlap with those involved in sequence production^53^. Such unresolved questions hinder attempts to determine if observed species differences in sequence learning reflect general cognitive constraints or the particular sensory, motor, and motivational features of different experimental paradigms^40,54^. We suggest that new approaches to the study of naturalistic behaviors, such as the action grammar extraction methods presented here, may help to address these issues.

Well-controlled artificial experimental paradigms will continue to be an essential tool for progress, but run the risk of producing results that are not generalizable to real-world behavior^17,18,55^. For example, motor sequence and statistical learning experiments to date have not addressed the complex actions (e.g. bimanual, multi-joint, transitive), reciprocal stimulus–response contingencies (action influences as well as being constrained by context), and multisensory (tactile, kinesthetic, auditory, visual) cue integration that are characteristic of real-world skills. These omissions will be problematic if sequence processing mechanisms are holistic and emergent rather than easily decomposable into modular components^55,56^. In the statistical learning literature, the paradoxical observation of similar behavior^54^ and partial neuroanatomical overlap^57^ for auditory vs. visual sequence learning combined with a lack of transfer, interference, or within-subject correlation across modalities^52^ suggests this may indeed be the case. Our method for extracting grammars from real-world behaviors addresses this issue by allowing us to quantify structural complexity while maintaining the irreducible complexity of the natural behavior. This creates the prospect of directly investigating the computational demands and neurocognitive substrates of complex sequential behaviors actually exhibited “in the wild” by both humans and non-humans.

In our chosen example of stone tool making, it is not possible to control lower-level kinematic, spatial, and visual features without altering the higher-level action structure that emerges from them and thus compromising the basis for analogy with real, archaeologically documented behaviors^58^. Grammar extraction allowed us to identify the abstract structure emerging from this low-level variability and to relate it back to particular goal-directed actions observable in the archaeological record. Specifically, we found variation in structural complexity to be driven by implementation of a technical operation known as platform preparation, which is important for establishing control over the size, shape, and location of detached flakes, and thus over the form of the finished piece^59^. The earliest currently documented instances of platform preparation date to approximately 500,000 years ago^28,60^ and may be related to the rapid encephalization that also occurred during the late Middle Pleistocene.

Structurally, platform preparation comprises a series of core rotations and repeated percussions embedded within a basic flake removal sequence^26,36^. Behaviorally, it requires adaptation of kinematic details (Fig. 1B) to different proximate objectives that are themselves defined by attention to subtle material properties of the core^61,62^ that determine fracture patterns. Neurophysiological experiments with artificial paradigms implementing such multiple, context-dependent stimulus–response rules reveal rostro-caudal gradients of abstraction in frontal cortex generally^63^, and in bilateral inferior frontal gyrus specifically for the selection of action chunks^50^. However, such studies employ discrete sets of simple, predefined perceptual stimuli and motor responses that do not require the multi-level extraction of units (segmentation) as well as relations between units (parsing) from a complex and continuous action stream as is characteristic of natural behaviors like stone tool making or language comprehension. Such issues of perceptual-motor complexity and temporal scale are increasingly relevant to debates about the neurocognitive mechanisms of structured sequence processing^64–66^.

**Figure 4:**
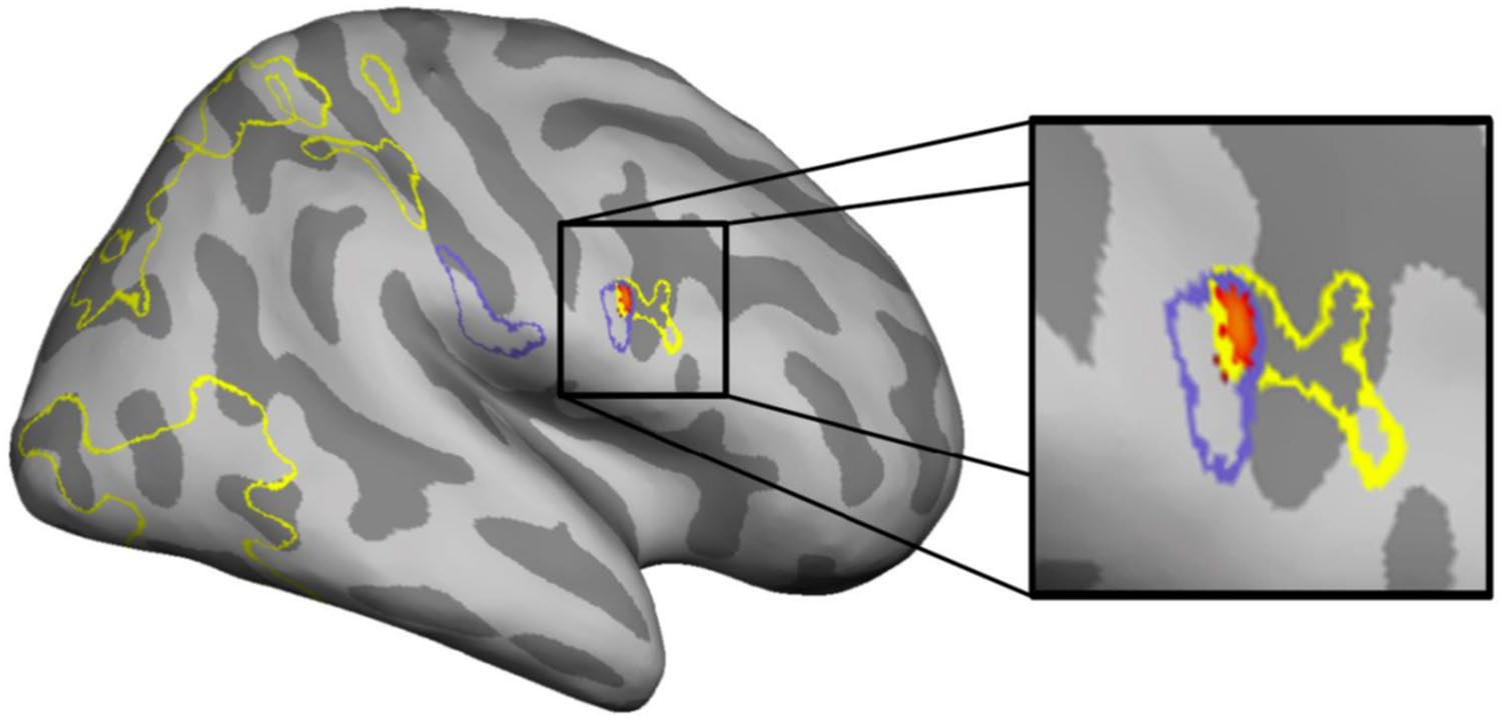
Covariance of BOLD response with tool-making stimulus structural complexity. Outlines corresponding to areas yielding significant correlation with the CFG (purple) and HMM (yellow) covariates describing action sequence complexity. Heatmap clusters represent the minimum of these two correlations where they overlap voxel-wise in the right pars opercularis of the inferior frontal gyrus (right).

To explore the potential application of our approach to neurophysiological research, we used HMM and CFG grammar extraction to measure the complexity of action sequences in Oldowan and Acheulean video stimuli from a published fMRI study of tool-making action observation^31^. To generate a continuous complexity measure from HMM, we used the difference in likelihood (measured by Akaike Information Criterion, see methods) between more (6 state) and less (4 state) complex models fit to the stimulus sequences. For CFG, we simply used the compression ratio. Results (Fig. 4) indicate that the two very different HMM and CFG metrics capture partially overlapping stimulus processing demands in the brain.

The conjunction of the two covariates reveals regions of left parietal operculum and rIFG (*pars opercularis)* that are specifically responsive to stimulus complexity irrespective of measurement method. This is consistent with prior evidence of inferior frontoparietal functional activation^31,32,67–69^ and structural remodeling^70^ in response to stone tool making, which have previously been interpreted using informal, qualitative estimations of technological complexity and reverse inference from published studies of other tasks. Parameterization of complexity through grammar extraction now allows direct identification of activity driven by sequence structure and more specific localization to cognitive control^71,72^ and multisensory integration^73^ regions of inferior frontal and parietal cortex. Together with structural and comparative evidence, this provides functional, archaeological, and neuroanatomical grounding for longstanding hypotheses of tool-language co-evolution^5,6,33–35,74^. Interestingly, the HMM covariate also picks up more widespread response across occipitotemporal and parietal portions of a “dorsal attention”^75^ or “multimodal integration”^73^ network. This suggests that the probabilistic, context-based description of structure provided by HMM may be a better fit to brain activity than the rule-based compression employed by CFG and raises the possibility that action grammar extraction could be used to test explicit hypotheses about neurocognitive mechanisms in the same way that has been done for language comprehension^76,77^.

The remarkable expressive power of human language derives from an ability to recombine a relatively small set of discrete units into a vast array of meaningful structures^3,29^. At the outset of the Cognitive Revolution, Lashley^2^ used the example of language to argue that *all* skilled behavior (and associated neural activity) is organized in this hierarchical fashion. This insight was subsequently applied to the specific case of stone tool making by Holloway^6^. Sixty-odd years later, however, we are still struggling with what Lashley identified as the “essential problem of serial order”: defining the “generalized schemata of action which determine the sequence of specific acts” that he termed “the syntax of the act.” Here we developed objective and generalizable methods for defining and quantifying these structures (action grammars) along with their neural correlates from raw behavioral data. While the detailed kinematics of hand actions to produce a tool vary from trial-to-trial considerably^27,42^, we found an invariant hierarchical structure underpinning performance at the symbolic level of actions. Our analytical approach does not postulate the existence of such “action grammars” a priori, but instead identifies them from raw behavioral data using machine learning techniques, showing that even with the same alphabet of actions qualitatively more complex artefacts can be produced by using measurably more complex action grammars.

This usefulness for analysis is complementary to our recent work on synthesis of behavior, i.e., when learning the generation of complex behavior in robotics and machine learning. In these artificial intelligence domains, we have shown that an action grammar approach to structure the learning algorithm’s representations of behavior does help speed up learning of complex tasks, it confers the ability to transfer learned skills between related tasks (which may often differ in kinematics but less so in their action grammars) and boosts the human-interpretability of the how a robotic system may plan and execute a complex task^78,79^.

Thus, our action grammar behavior analysis approach’s promises not only broad utility for behavioral and social sciences, but also the finding that our automatic identification of action grammars maps to distinct neural correlates offers the potential for novel quantitative approaches to study hierarchical structure of complex behavior with benefits across many applications from dexterous prosthetics^80^, the training of complex skills in surgery^81^ or sports^82^ to human-like artificial intelligence.

## Methods

Methods for this study have previously been made available as a preprint^83^ and are reproduced here with minor clarifications here. De-identified data, descriptors, and code sufficient to produce results published here are available on the FigShare community archive https://doi.org/10.6084/m9.figshare.14703687.v1.

### Tool replication

Tool making videos analyzed for this study were produced by video recording two research participants experimentally replicating Paleolithic tool forms. This research was approved by the University College London Research Ethics Committee [0603/001], all experiments were performed in accordance with relevant guidelines and regulations, participant identities were not revealed, and each participant provided written informed consent. Participants were both expert stone tool makers with decades of experience. These video-recorded replication experiments included 9 instances of Oldowan knapping and 8 instances of Acheulean knapping. In each experiment, a piece of flint was worked until either completely exhausted (Oldowan) or successfully shaped into a refined handaxe (Acheulean). Six of the handaxes produced in these experiments have previously been described and compared to archaeological examples for the Middle Pleistocene site of Boxgrove^28^. Kinematics from a different subset of the experiments (3 Oldowan, 2 Acheulean) have been published^27^.

Experimental replication is a long-established research method in archaeology, especially with respect to flaked stone technology^58^. Our tool-making experiments drew upon this background to model simple flake production (cf. “Oldowan”, “Mode 1”, “Mode C”^84^, here termed "Oldowan" ) and refined handaxe shaping (cf. “Later Acheulean handaxe”, “Mode 2”, “Mode E2”^84^, here termed "Acheulean"). Previous experimentation has shown that a wide range of Oldowan forms may be replicated through hard-hammer free-hand percussion without intentional core shaping^85^, whereas other techniques (e.g. bipolar, passive hammer) produce diagnostic traces that are less common in the archaeological record^86^. Although there is some evidence of structured reduction strategies in the Oldowan (e.g. preference for unifacial vs. bifacial flaking^87,88^) it is possible to produce most or all Oldowan forms through unstructured (cf. “mindless”^89^ or “least effort”^85^) flaking. We thus instructed our subjects to knap Oldowan experiments in an opportunistic fashion, following the definition of “simple debitage” provided by^90^. For Acheulean experiments, subjects were instructed to produce “refined” Acheulean handaxes of the kind known from the site of Boxgrove (with which subjects were familiar). This included the use of soft hammers and simple platform preparation (faceting), both of which are attested in the Boxgrove archaeological collection^28^. Experimental handaxes produced were comparable in refinement and debitage morphology to those from Boxgrove^28^.

Paleolithic tool making occurred over a vast time period and many millions of square miles and encompasses substantial variation that could not be included in our experiments. The methods we did select are considered broadly representative of early and late Lower Paleolithic technology, and details of the production techniques employed match those documented in specific archaeological collections. We thus consider our training protocol to be both generally representative and specifically accurate in re-creating Paleolithic tool-making action sequences.

### Event coding

We defined an action alphabet consisting of 7 event types encompassing the elementary body movements and object transformations present in every sequence of both technologies. Events were transcribed from video-recordings using Etholog 2.25^91^. Events were defined as follows:

- Percussion: Striking core with percussor (hammerstone or antler billet).
- Target Change: A change in the location of percussion on the core.
- Grip Shift Core: Repositioning of the hand grasping the core.
- Grip Shift Tool: Repositioning of the hand grasping the percussor.
- Inversion: Flipping over the core without otherwise reorienting.
- Flake detach: Removal of a flake (judged to be) > 20 mm.
- Tool Change: Exchange of one percussor for another.

This provides a minimalistic alphabet intentionally designed to limit the need for subjective interpretation and to avoid building prior hypotheses into the coding scheme. In particular, any attempt to infer the intention of the knapper (e.g., identifying a flake detachment as “preparatory” or “thinning”) was avoided. Much richer description of knapping actions in terms of technological function is both possible and informative^25,92^, but was not in line with our aim to develop a data-driven and generalizable method. The coding scheme was developed through pilot work with the MRI stimulus videos (Table 1 in^31^) and the new replication experiments reported here to be complete (every action on the core or percussor is coded), exclusive (no action could have two codes), and unambiguous. During this pilot work, we removed and/or clarified the criteria of any codes that generated uncertainly or inconsistency during trial coding.

For example, we initially recorded an eighth event type, “Light Percussion” (cf. Fig, 1B), which was not subsequently employed in analysis. This event was defined as “Striking core with percussor using small amplitude arm movements not intended to detach flakes > 20 mm” and was omitted because: 1) it required interpretation, 2) it did not occur in Oldowan sequences, and 3) it might be ambiguous with the Percussion event. Thus, we treated all instances of “light percussion” simply as percussion. However, this gesture—typical of a technical operation known as “striking platform preparation”—was rediscovered by our HMM and Sequitur analyses based purely on sequential structure analysis, thus providing a validation of our iterative approach to developing a reliable and unambiguous ethogram and of our data-driven analytic approach. While the actual alphabet used here is specific to stone tool making, this approach to coding could be generalized to any sequential behavior.

### Hidden Markov modeling

We fit Hidden Markov Models (HMM) to the action sequences using the Baum-Welch algorithm implemented in Kevin Murphy’s Bayes’ Net Toolbox. As the algorithm is very sensitive to the initial estimates of the transition and emission matrices, we fit each data set 1000 times for each number of states by randomly varying the initial condition and only picked the HMM with the highest log-likelihood. To compare HMMs with different number of hidden states with each other, we computed the Bayesian Information Criterion (BIC) which gives a measure of model fitness penalised by the number of free parameters in the model.

From the 6 state Acheulean HMM we obtained the most likely state-sequences through the Oldowan and Acheulean action sequences by using the Viterbi algorithm. To investigate whether the obtained hidden state sequence, contained more structure, we fitted a second, 2-state HMM onto the state sequences. As previously described, 1000 runs were performed to obtain the best-fitting HMM. Using the Viterbi algorithm again gives rise to a hidden state sequence within the hidden state sequences, a hidden “States of States” (SoS) sequence.

### Deterministic context-free grammar fitting

Any stochastic regular grammar can be represented by a uniquely corresponding HMM where outputs correspond to terminal symbols. Left regular stochastic grammars—because they are strictly equivalent to first order Hidden Markov Models—can only model phenomena with very short memory. Stochastic Context-Free Grammars represent a super-set of stochastic grammars which can feature long term memory and very hierarchical organization.

Sequitur^41^ is a recursive algorithm that infers a hierarchical structure in the form of a context-free grammar from a sequence of discrete symbols. We chose Sequitur because we are concerned here with the smallest grammar problem (simplest explanation according to Occam’s Razor) which has been applied to musical scores, DNA sequences and are, due to their simple nature, very data efficient^93^. Other methods are based on data compression approaches which are difficult to apply for short sequences (such as our ethogram data) due to these methods having fixed and data-size dependent information that need to be represented, but the fixed size components outweigh the benefits of the variable length representation^94–96^.

The Sequitur algorithm constructs a grammar by substituting repeating symbol digrams in the given sequence with new rules and therefore produces a concise representation of the sequence. The algorithm works by scanning a sequence of terminal symbols and building a list of all the symbol pairs which it has read. Whenever a second occurrence of a pair is discovered, the two occurrences are replaced in the sequence by a non-terminal symbol, the list of symbol pairs is adjusted to match the new sequence, and scanning continues. If a pair's non-terminal symbol is used only in the just created symbol's definition, the used symbol is replaced by its definition and the symbol is removed from the defined nonterminal symbols. Once the scanning has been completed, the transformed sequence can be interpreted as the top-level rule in a grammar for the original sequence. The rule definitions for the non-terminal symbols which it contains can be found in the list of symbol pairs. Those rule definitions may themselves contain additional non-terminal symbols whose rule definitions can also be read from elsewhere in the list of symbol pairs.

For example:

Input sequence: *the little cat chases the mouse the little cat catches the mouse the big cat chases the little cat the little cat runs away from the big cat*

Compressed sequence: *r2 chases r3 r2 catches r3 r5 chases r2 r2 runs away from r5*

Grammar:

- *Root -*> *r2 chases r3 r2 catches r3 r5 chases r2 r2 runs away from r5*
- *r2 -*> *the little cat (used 4 times)*
- *r3 -*> *the mouse (used 2 times)*
- *r5 -*> *the big cat (used 2 times)*

We ran Sequitur on each sequences in both the Acheulean and the Oldowan data sets and enumerated all the rules found across both data sets. After inferring rules from the combined Acheulean and Oldowan data set, we found that some rules only occurred in Acheulean sequences (Supplementary Fig. S2).

The Sequitur algorithm reduces the length of the sequences by replacing terminal symbol strings with aggregating rule strings. This compresses the sequence by reducing its redundancy. Figure 3G shows that sequences in our Oldowan and Achuelean samples share common compressible structure within samples but are distinct across samples. This is indicated by the fact that their pre and post-compression lengths are linear and have distinct slopes. Linear regression fit for Acheulean data is R^2^ = 0.9852 with slope = 0.13; for Oldowan data R^2^ = 0.9982 and slope = 0.34. The inverse slope on this plot corresponds to the data compression rate through rule extraction.

Sequitur as a compression algorithm is loss-less, in that reverse applying the rules recovers the original sequence error free, and thus the same information is communicated by fewer symbols. This contrasts with the hidden states of the HMM that only capture probabilistically a higher order structure. A Sequitur compressed sequence must have more information per character and this gain in information density can be quantified using Shannon’s entropy measure. Shannon’s entropy is computed directly as the log probability of each symbol averaged over all symbols. A sequence with equally probable use of all symbols has the highest entropy, while a sequence using only a single symbol has an entropy of 0. Entropy thus measures how unpredictable a symbol is. We plotted the pre and post compression entropies in Fig. 3H. Pre-compression entropy of Acheulean sequences is considerably lower than that of Oldowan sequences due to the much higher frequency of percussion events. However, post-compression entropy is considerably higher for Acheulean sequences than Oldowan sequence. Thus, pre-compression Acheulean elements (rules + symbols) carry less information than Oldowan elements whereas after compression the reverse is true.

### fMRI covariates

In order to generate covariates for fMRI analysis it was necessary to produce continuous measures of complexity for the 20 s video stimuli. For HMM, we first applied the method described above to each stimulus and then evaluated how well the stimulus was explained by the two respective (Acheulean 6 hidden state vs. Oldowan 4 hidden state) HMM models. Sequence length was both short and variable (stimuli were controlled for time rather than number of actions), so we employed the Akaike Information Criterion [AIC] which, unlike BIC, is not directly dependent on sample size in order to avoid confounding sequence length with model likelihood. Differences in AIC between models indicate the relative strength of evidence in their favor. Because our models differ in complexity, this difference provides a continuous measure of how complex (i.e. Acheulean-like vs. Oldowan-like) each short stimulus sequence is compared to models derived from our entire corpus. As a lower AIC indicates a more probable model, decreasing values for Acheulean – Oldowan AIC indicate increasing stimulus complexity and we predict a negative correlation with BOLD response measured by fMRI (cf. Figure 4).

For CFG, we applied the same deterministic grammar extraction approach discussed above. However, in our main analysis, sequitur was applied separately to each sequence. To generate a CFG covariate comparable to our HMM AIC metric, it was necessary to generate a global set of rules derived over the entire corpus to which individual stimuli could be compared. We thus fitted sequitur to the complete set of all sequences in one run. This provided us with a sequitur parse using compressed rules for the entire corpus. We then broke down the compressed rules and matched them to the individual stimulus sequences and computed the basic metrics (as for the long sequences) for these matched compressed sequences. The compression ratio for each stimulus provides a straightforward measure of complexity, we used post- over pre-compression values so that our CFG metric would parallel our HMM metric in matching decreasing values with increasing complexity and predicting negative correlation with BOLD.

### fMRI analyses

Experimental paradigm and participants were presented ref.^18^. Briefly, 10 Naïve, 10 Trained and 5 Expert subjects observed 20-s videos of an expert demonstrator performing two tool-making methods of differing complexity and antiquity: the simple ‘Oldowan’ method documented by early tools 2.5 million years ago; and a more complex ‘Late Acheulean’ method used to produce refined tools 0.5 million years ago. In the present SPM analysis, the two categories of tool making were defined as two conditions, and complexity scores (HMM and CFG) were added as covariates describing each stimulus in two individual subject analyses.

The effect of these covariates combined across the two categories of stimuli were entered in two multisubject analyses across the 20 non-expert participants, thresholded at *p* < 0.05 FDR-corrected at the cluster level (Fig. 4). Experts were omitted due to a small sample size insufficient to properly assess confounding expertise and automaticity effects^64,65^. To confirm the overlap in left parietal and right frontal cortices between the two analyses, a conjunction (“&”) was calculated between the T-maps describing the voxels yielding significant negative correlation with the two covariates. This analysis yielded two clusters, one in the left parietal operculum and one in the posterior part of the right inferior frontal gyrus corresponding to the pars opercularis according to the Anatomy toolbox^97^.

## Supplementary Information

Supplementary Figures.

## Data Availability

De-identified data, descriptors, and code sufficient to produce results published here are available on the FigShare community archive https://doi.org/10.6084/m9.figshare.14703687.v1.
