# Supplementary material for: The measurement, evolution, and neural representation of action grammars of human behavior
Source: Sci Rep. 2021 Jul 2;11:13720. doi: 10.1038/s41598-021-92992-5 (PMC8253764; doi:10.1038/s41598-021-92992-5)
Supplement: Supplementary file 1 — Supplementary Figures. [file 41598_2021_92992_MOESM1_ESM.pdf]

Supplementary Information

Action Grammars of Human Behavior – Measurement, Evolution, and Neural Representation

Dietrich Stout[1], Thierry Chaminade[2], Jan Apel[3] & A. Aldo Faisal[4,5,6,7]

[1]Department of Anthropology, Emory University, Atlanta, GA, USA

[2]Institut de Neurosciences de la Timone, Aix Marseille Université, Marseille, France

[3]Department of Archaeology, Stockholm University, Stockholm, Sweden

[4]Department of Bioengineering, Imperial College London, London, United Kingdom

[5]Department of Computing, Imperial College London, London, United Kingdom

[6]Integrative Biology, MRC London Institute of Medical Sciences, London, UK

[7]Behaviour Analytics Lab, Data Science Institute, London, UK

Supplementary Figures 1-3

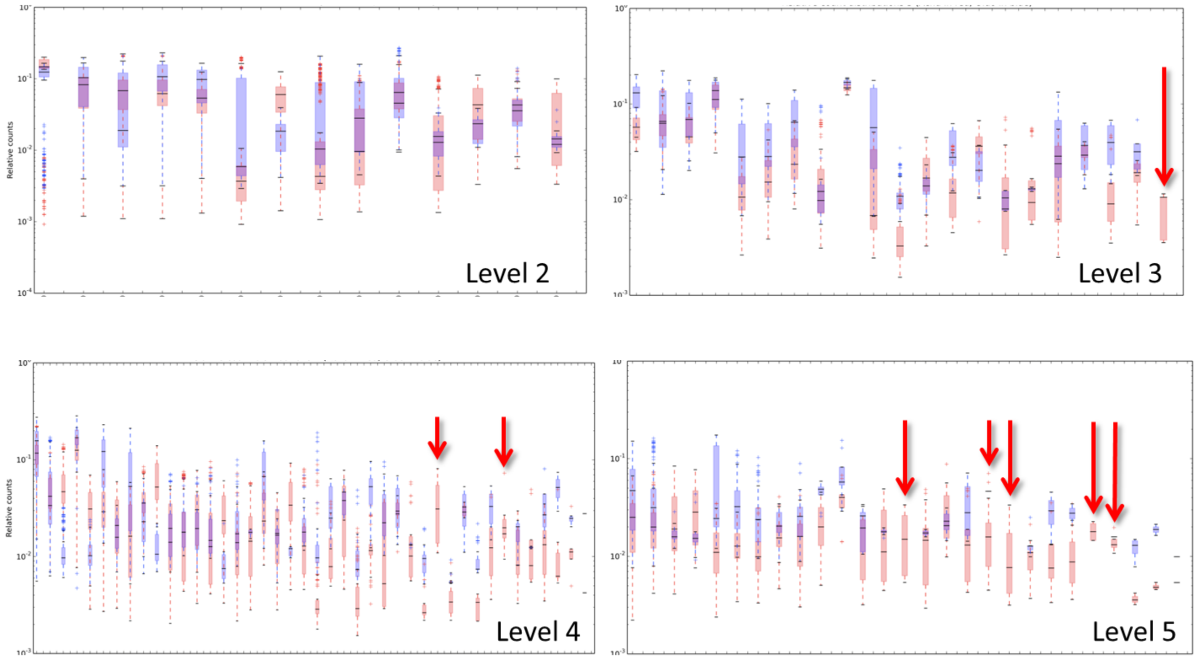

Supplementary Fig. 1. Relative frequency of rule appearance across all Oldowan (blue) and Acheulean (red) sequences. Red arrows indicate rules occurring only in Acheulean sequences.

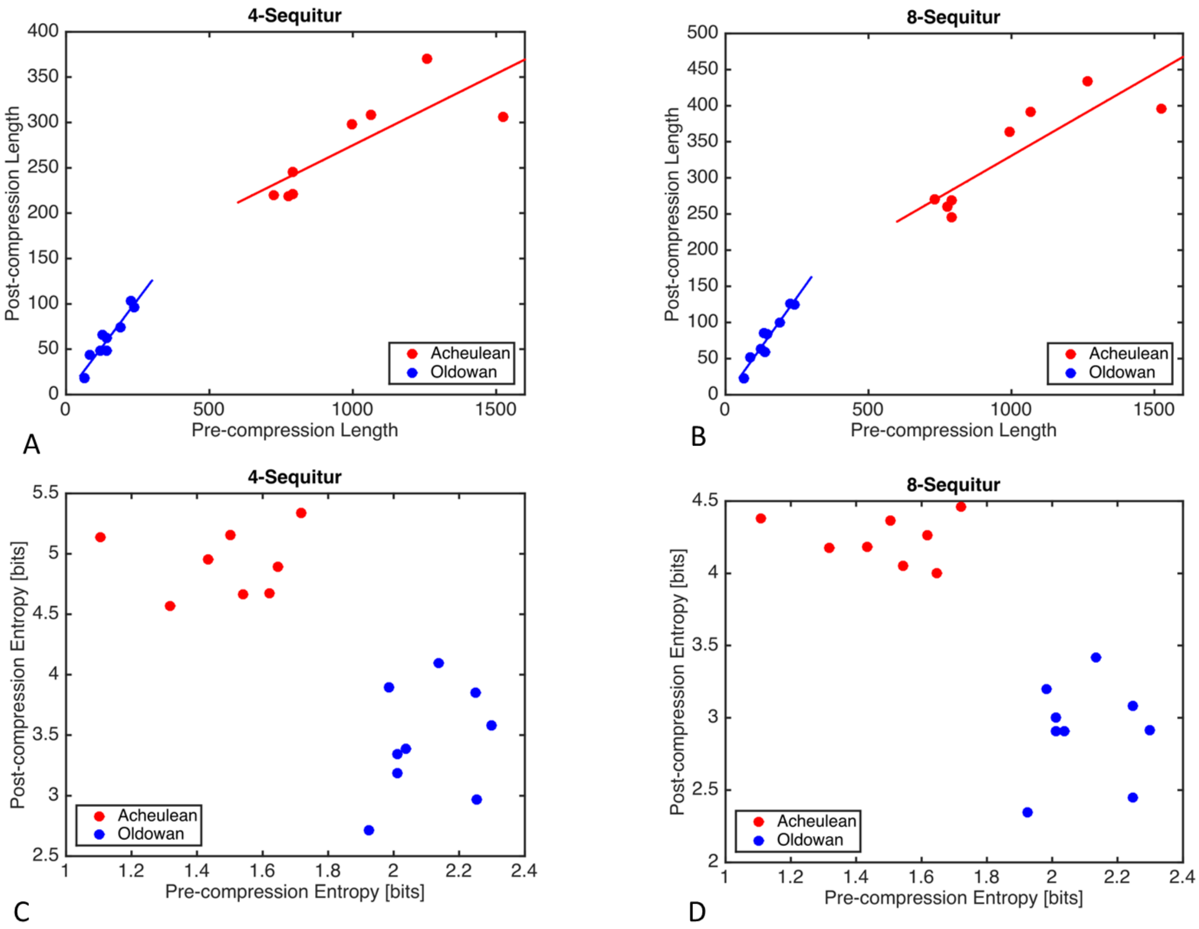

Supplementary Fig. 2. Effect of k-Sequitur compression on sequence length **(A, B)** and entropy **(C,D)** for increasing values of k.

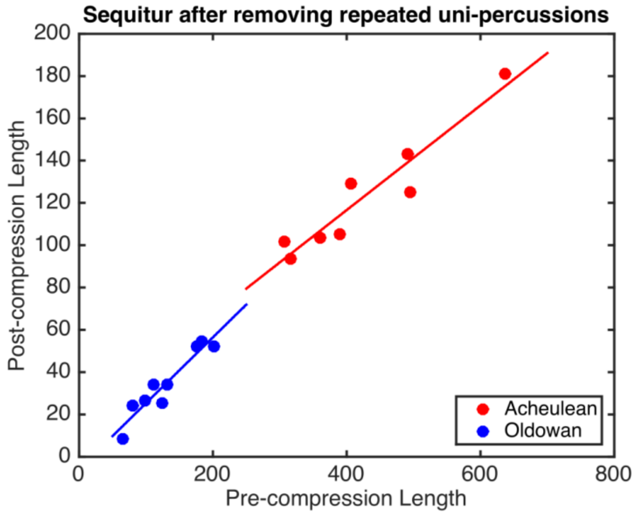

Supplementary Fig. 3. Effect of Sequitur compression after removing repeated percussions.
